# Recovr reality - Recover after injury or surgery to the brain and spinal cord with virtual Reality: ideal stage 2a clinical feasibility study

**DOI:** 10.1186/s12984-024-01499-3

**Published:** 2025-03-03

**Authors:** William Stephen Bolton, Rohitashwa Sinha, Sara Cooper, Oluwaseyi Adebola, Elisa Stephenson, Seonaid Ewan, Rachel Hunsley, Victoria Kearton, David Stevens, Ryan Koshy Mathew

**Affiliations:** 1 Department of Neurosurgery, Leeds Teaching Hospitals NHS Trust, London, UK; 2 School of Medicine, University of LeedsTrust, London, UK; 3 Centre for Immersive Technologies, University of Leeds, London, UK

## Abstract

**Aim:**

Neurorehabilitation is fundamental to improve outcomes for patients recovering from injury to the central nervous system. Access to neurorehabilitation is limited by resource shortages; the consequences of which are unfulfilled therapy needs, longer hospital stays and detrimental effects on quality of life. Virtual reality (VR) could be used to enhance neurorehabilitation in a self-directed, safe, virtual environment. The aim of this study is to investigate the feasibility of a VR rehabilitation programme in an acute neurosurgical inpatient environment to improve neurorehabilitation.

**Method:**

A single-group, prospective, clinical feasibility study was conducted in a tertiary UK neurosurgical department. The study included patients aged 16 and over who had neurosurgical care following surgery or traumatic brain or spinal cord injury. Participants were offered a VR session at least once per day for the first 14 days post-surgery/injury or until discharge, whichever came first, with reasons for non-engagement collected. Primary outcomes were feasibility outcomes and secondary outcomes included rehabilitation engagement.

**Results:**

Of the thirty-nine eligible participants approached, thirty-two participants were recruited and received VR at least once. Intervention fidelity was deemed a success, as none of the VR equipment or applications failed. Median time between injury or surgery and first VR use was three days (IQR = 8.25). The Hopkins Rehabilitation Engagement scale and Simulation Sickness Questionnaires were deemed feasible instruments to measure outcomes.

**Conclusions:**

We confirmed feasibility of using a VR rehabilitation tool for neurosurgical patients in this study. This now facilitates progression to a multi-centre, prospective, randomised, controlled, unblinded, parallel-group trial of VR-enhanced neurorehabilitation versus standard neurorehabilitation for improving recovery after neurotrauma or neurosurgery.

## Introduction

Brain and spinal cord injuries are a leading cause of death and disability in the world [1]. Traumatic brain injury (TBI) affects 55 million people and spinal cord injury (SCI) affects 27 million people worldwide, costing over £350 billion per year in direct care costs and lost economic output [1]. For those who survive, recovery is often incomplete and protracted and lifelong care is required for many. In 2016 TBI caused 8.1 million years lived with disability and SCI caused 9.5 million years [1]. The socioeconomic impacts of TBI and SCI in the UK are substantial, costing the UK economy approximately £17 billion per year [2, 3]. These figures encompass the costs associated with lost work, health and social care needs, and premature mortality. Following acute stabilisation and surgical intervention, neurorehabilitation is started to maximise functional recovery.

Furthermore, a period of neurorehabilitation is often required following cranial or spinal neurosurgery for non-traumatic pathologies. In the UK the mean length of stay for patients who had elective or non-elective cranial neurosurgery is 5.8 and 19.4 days respectively, with an average of 14% of patients spending more than 28 days with the neurosurgery provider [4]. Although neurorehabilitation continues as required in other specialist healthcare settings, a recent analysis revealed that the waiting list for neurosurgery is so significant that a national seven-day tertiary neurosurgical service would require 18 months to clear the backlog [5]. Therefore, technology that can accelerate recovery after surgery, facilitating faster discharge from acute hospital or return to independence, would have a significant impact on this incredibly costly problem for patients, society and the healthcare system in general.

Clear evidence exists for early and intense neurorehabilitation leading to better functional outcomes in neurosurgical trauma and cancer patients [6, 7]. A challenge is the limited amount of neurorehabilitative human resource to address this patient cohort, along with space, availability of enriched environment, and provision of novel tasks. Virtual reality (VR) is a type of digital immersive technology using spatial computing where the user interacts with a programme through a head mounted display, or headset, with hand-held controllers. The technology has been used in conjunction with neurorehabilitation experts after stroke and spinal cord injury for both physical and cognitive recovery, and there is evidence of safety and effectiveness at improving the speed and quality of recovery [8–10].

Specific to this study, it is reasonable to consider whether the technology would provide similar benefits for patients after neurosurgery or other types of neurotrauma. There are unique patient specific safety and feasibility considerations in this cohort that are not fully investigated in the current literature, such as the impact of wearing a headset after undergoing craniotomy and the acceptability of the technology to patients with serious cancer diagnoses or sudden traumatic injuries. Furthermore, evidence suggests that initiating intense and frequent rehabilitation early in the acute phases post injury can leverage a window of opportunity in terms of improved neuroplasticity which is essential for maximising recovery [11]. Therefore, the aim of this study was to investigate the feasibility and initial clinical safety of using VR in the neurorehabilitation process for acute neurosurgery and neurotrauma patients in a tertiary acute care setting.

## Methods

### Study design

A single group, clinical feasibility study was designed at a large tertiary neurosurgical centre in the United Kingdom. Eligibility criteria were as follows:

Inclusion criteria:

- Adults aged 16 years and older with no upper age limit.
- Willing and able to provide written informed consent.
- Having received neurosurgery for neuro-oncology OR neurotrauma OR conservative management for neurotrauma on a level 1 ward.
- Able to wear the physical head mounted display device e.g. a large craniectomy prevented head mounted display use.

Exclusion criteria:

- Participants unwilling or unable to provide written informed consent.
- Participants with photosensitive epilepsy.

Ethics approval was provided by the Health Research Authority (22/NW/0061) and the Leeds Teaching Hospital NHS Trust (IRB approval n. 295487). Participants were recruited from January to September 2023. The study was designed in accordance with the IDEAL Framework’s guidance on evaluating innovation for Stage 2a studies [12]. As such, no formal sample size is required for feasibility studies. However, literature values reveal a sample of approximately 30 participants is deemed sufficient to evidence feasibility outcomes [13]. This was set as the recruitment target for this study. The reporting followed guidance from the 2016 CONSORT extension statement and a subsequent editorial for non-randomised feasibility studies [14, 15]. To evaluate the methodological feasibility of VR neurorehabilitation in this context, a series of 14 study design elements were considered in line with practice seen in the literature [16, 17].

## Experimental protocol

The VR intervention used hardware in the form of PICO Neo3 VR headsets (Developed by ByteDance, Beijing, China). Each headset was connected to two hand controllers which could be used if the participant was doing a physical therapy task. If they were unable to use the hand controllers, or wanted to just do a cognitive therapy exercise, then they could use the headset alone. Visual impairment itself was not a preclusion to involvement in the study, and participants could wear glasses under the headset or / and the headset’s focal point could be mechanically adjusted to suit. The software used in the intervention was from SyncVR Medical (Amersfoort, The Netherlands) who provide a number of rehabilitation applications: the two used in this study were SyncVR Fit (version 334) for physical rehabilitation which provides a range of motor tasks that can be performed with upper and lower limbs, lying, seated, or standing, to accommodate the participants needs and desires; and SyncVR Relax & Distract for cognitive rehabilitation (version 20500) which provides a range of mental exercises through visual and auditory stimulation such as simulating relaxing environments and breathing exercises. The hardware was cleaned with a clinically approved wipe before and after each use. Participants who had head wounds additionally wore a surgical scrub cap over their dressings before using the headset.

When deemed ready for rehabilitation after injury or surgery as per standard practice in level 1 wards (i.e. not intensive/critical care), consented participants underwent a brief (approximately 5–10 min) educational VR session where they were instructed by members of the research team on how to use the device and applications. Inpatient neurorehabilitation physiotherapists then facilitated a VR session with an application chosen based on patient preference. The applications could be used in sitting, standing, or lying down positions and with none, one or both hand controllers. VR sessions were conducted at the patient’s bedside or in a physiotherapy area during their normal rehabilitation sessions under the supervision of the neurorehabilitation team. All participants received the standard of care, and this VR session did not replace any existing therapy sessions. Patients used the VR device for as long as they wished, up to a maximum of 30 min, and were then asked to complete data collection instruments.

## Data collection and statistical analysis

The primary outcomes were feasibility outcomes of participant recruitment (number of patients approached versus number recruited), intervention fidelity (incidences of intervention technical failure and reasons) and intervention acceptability (withdrawal from study or intolerance of the VR session and reasons). These outcomes were evaluated using study-specific data collection forms and validated instruments completed (1) immediately after the VR session and (2) upon discharge from the neurosurgical centre. Validated instruments used were the System Usability Scale to evaluate participant experiences of using the technology, the Simulation Sickness Questionnaire to screen for side-effects of VR (both given after the VR session) and the Hopkins Rehabilitation Engagement Rating Scale, which is a therapist-completed questionnaire that evaluates the patient’s engagement in their rehabilitation (completed at discharge) [18–20]. Detailed information on neurological deficits were not collected because no effectiveness outcomes were included. All included patients had a need for neurorehabilitation, and only information about significant neurological impairment such as at least one limb weakness of power grading 3/5 or worse or a visual field defect / inattention were collected. Information on adverse outcomes, such as wound infection, seizures and injury related to rehabilitation, within the first 30 days after VR use were collected for every participant. Data was tabulated, descriptive statistics were performed, and charts were produced using Microsoft Excel (version 16.83).

## Results

### The feasibility and acceptability VR neurorehabilitation in acute neurosurgical settings

**Fig. 1:**
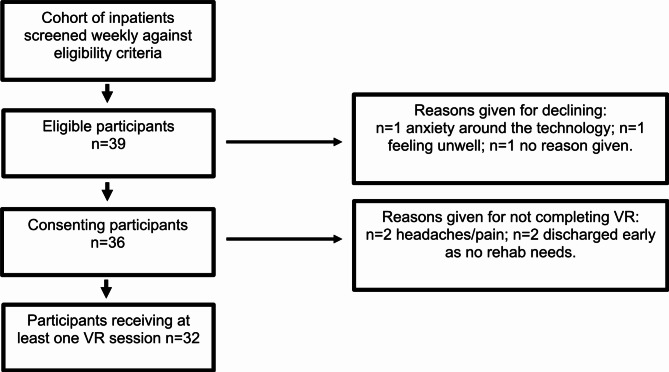
CONSORT participant recruitment flow diagram

Figure 1 displays the CONSORT participant recruitment flow diagram. Of the 39 participants who were deemed eligible and approached, 36 consented to be recruited into the study (a recruitment rate of 92%) and *n* = 32 went on to complete a VR session (an intervention completion rate of 88%). Reasons given for declining to participate are presented in a flow diagram (see Fig. 1). These included anxiety around the technology, feeling unwell and having headaches. Table 1 summarises the patient characteristics. Two-thirds of patients (*n* = 21; 66%) were post-operative neuro-oncology patients, the remaining one-third had neurotrauma with a mixture of brain or/and spinal cord injuries. The mean age was 56 years, and the oldest participant was 89 years, suggesting that advanced age need not be a barrier to engaging with the technology. There were proportionally more males, with a male to female ratio of 1.5:1. VR was most frequently initiated on the second day post injury/surgery and a spread of cognitive and physical rehabilitation apps were utilised with more participants choosing cognitive options (Table 2).

**Table 1:** Patient demographics

Variable	Value
Age (mean ± SD)	56 (± 19)
M	21:14
Post op tumour	21 (66%)
TBI (Conservative Management)	5 (16%)
SCI (Post operative)	1 (3%)
SCI (Conservative Management)	4 (13%)
Both TBI and SCI (Conservative Management)	1 (3%)
At least one limb 3/5 weakness pre-intervention	12 (38%)
Visual field defect or inattention	6 (19%)

**Table 2:** VR intervention details

VR app used	Frequency
Sync VR Fit	6
Relax & Distract	16
Both	10
Mean VR session duration (minutes)	10
First VR session use most frequently on day 2 post injury / surgery	

**Table 3:** 30-day intervention-related adverse events and VR side-effects as assessed by the Simulation Sickness Questionnaire

Adverse outcomes at 30 days				
Seizures	0			
Wound infection	0			
Rehab related injury	0			
SSQ Symptom n= (%)	**None**	**Slight**	**Moderate**	**Severe**
1. General Discomfort	28 (88%)	2 (6%)	2 (6%)	0 (0%)
2. Fatigue	32 (94%)	2 (6%)	0 (0%)	0 (0%)
3. Headache	28 (88%)	4 (13%)	0 (0%)	0 (0%)
4. Eye Strain	28 (88%)	4 (13%)	0 (0%)	0 (0%)
5. Difficulty Focusing	25 (78%)	4 (13%)	3 (9%)	0 (0%)
6. Salivation Increasing	26 (81%)	0 (0%)	0 (0%)	0 (0%)
7. Sweating	32 (100%)	0 (0%)	0 (0%)	0 (0%)
8. Nausea	30 (94%)	2 (6%)	0 (0%)	0 (0%)
9. Difficulty Concentrating	31 (94%)	1 (3%)	1 (3%)	0 (0%)
10. Fullness of the Head	27 (84%)	4 (13%)	1 (3%)	0 (0%)
11. Blurred Vision	25 (78%)	7 (22%)	0 (0%)	0 (0%)
12. Dizziness with Eyes Open	29 (91%)	3 (9%)	0 (0%)	0 (0%)
13. Dizziness with Eyes Closed	32 (100%)	0 (0%)	0 (0%)	0 (0%)
14. Vertigo	32 (100%)	0 (0%)	0 (0%)	0 (0%)
15. Stomach Awareness	31 (94%)	1 (3%)	0 (0%)	0 (0%)
16. Burping	32 (100%)	0 (0%)	0 (0%)	0 (0%)

**Table 4:** Summary of findings against 14 methodological issues for feasibility research

Methodological items	Findings	Evidence
1. What factors influenced eligibility and what proportion of those approached were eligible?	A number of patients who lacked capacity were ineligible in this study. In future studies, deferred consent may be beneficial as a lot of these patients are still able to engage in neurorehabilitation.	36 agreed to consent, only three screened declined to consent. More may have consented if sample size had been larger.
2. Was recruitment successful?	Yes. Recruiting success is often defined as 80% of eligible participants agreeing and being recruited into the study.	32 out of 39 (82%) eligible participants agreed to take part and were recruited and underwent the intervention.
3. Did eligible participants consent?	Yes. The majority of participants agreed to consent.	Only 3 did not wish to consent.
4. Were participants successfully randomised?	No randomisation in this study.	No randomisation in this study.
5. Were blinding procedures adequate?	No blinding in this study.	No blinding in this study.
6. Did participants adhere to the intervention?	Yes. Successful adherence to the intervention is often defined as at least 80% of participants successfully completing the intervention.	32 out of 36 (89%) participants completed the VR intervention.
7. Was the intervention acceptable to the participants?	Participants were keen to engage with the VR intervention and a majority perceived it as acceptable and useful.	Figs. 2 and 3 summarise positive acceptability indicators.
8. Was it possible to calculate intervention costs and duration?	No economic evaluation in this study.	No economic evaluation in this study.
9. Were outcome assessments completed?	Yes, the instruments used were completed.	100% instrument completion rate if VR intervention was undertaken.
10. Were outcomes measured those that were the most appropriate outcomes?	All outcomes were deemed valid and appropriate.	Participant-completed forms were largely complete (missing data points in 7 instances)
11. Was retention to the study good?	Successful retention is often defined by less than 10% attrition rate.	All participants who completed VR intervention completed the data collection instruments.
12. Were the logistics of running a multi-centre trial assessed?	No. This was a single-centre feasibility study.	No. This was a single-centre feasibility study.
13. Did all components of the protocol work together?	The components of the study and the intervention itself worked in this feasibility study	Adherence to the intervention and study processes met the accepted feasibility criteria and show feasibility of progressing to a larger study.
14. Did the feasibility/pilot study allow a sample size calculation for the main trial?	No. A sample size for a future full RCT was not calculated from the data in this study.	No. A sample size for a future full RCT was not calculated from the data in this study.

### The safety and fidelity of VR neurorehabilitation in acute neurosurgical settings

Table 3 highlights that there were no adverse outcomes in terms of seizures, wound infections or injuries related to VR rehabilitation in the first 30 days after use, demonstrating strong early safety data. Table 3 describes the incidence of side-effects of VR as assessed by the Simulation Sickness Questionnaire. No participants reported severe side-effect symptoms, but almost a quarter reported slightly blurred vision at times during using the VR headset. Table 4 summarises the response to 14 methodological considerations in feasibility research that are used to assess feasibility of study design and delivery.

## Motivation to engage with VR neurorehabilitation

**Fig. 2:**
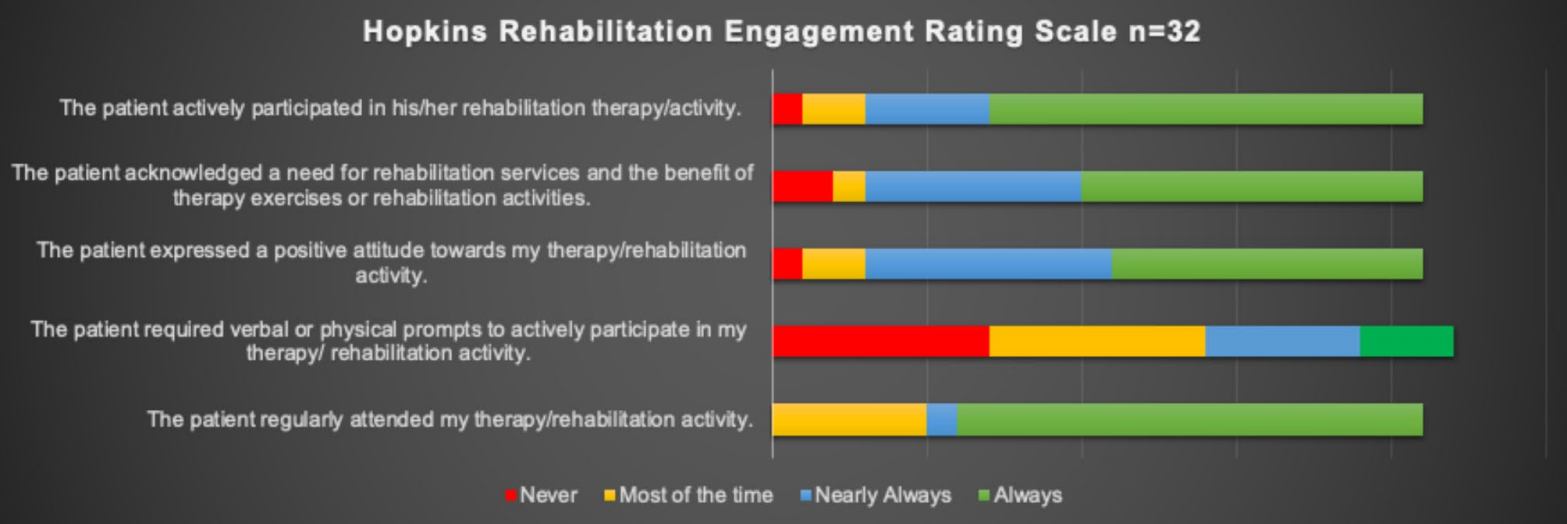
Hopkins Rehabilitation Engagement Rating Score for participants

**Fig. 3:**
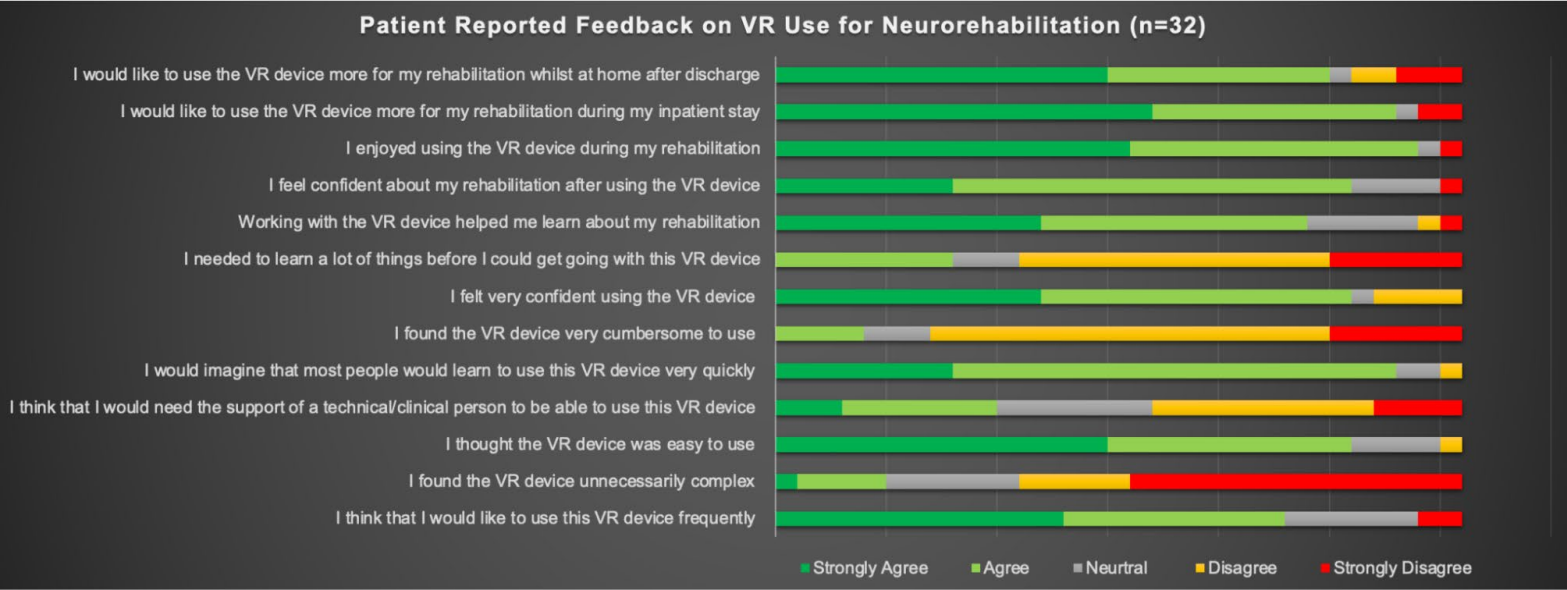
Patient Reported Feedback on VR Neurorehabilitation

Overall engagement was high. Figure 2 displays participants’ engagement in their neurorehabilitation as assessed by their therapists. Participants were also asked to complete a study-specific data collection instrument including a System Usability Scale and provided largely positive feedback on the use of VR in their neurorehabilitation. The majority agreed or strongly agreed that the VR intervention was acceptable and perceived as useful for their recovery. Indeed, the majority expressed the view that they would have liked more VR as an inpatient and would have used this as an outpatient to continue their neurorehabilitation in other settings, such as at home. Details of these results are visualised in Fig. 3.

## Discussion

This IDEAL Stage 2a feasibility clinical study has demonstrated that VR neurorehabilitation in an acute neurosurgical setting for neuro-oncology and neurotrauma patients is feasible, safe, and acceptable to deliver with high fidelity. Side-effects were minimal and tolerable for the majority of patients. This early safety profile supports the feasibility of VR integration in acute neurosurgical rehabilitation settings, although larger powered clinical effectiveness studies, with health economics evaluations, longer follow-up, and patient centred endpoints such as composite physical, cognitive and quality of life metrics.

Though the study observed minimal VR side-effects, the most common adverse experiences included mild symptoms like blurred vision. Similar studies of VR use in neurorehabilitation report comparable rates and attribute side-effects to post-operative factors such as residual anaesthesia effects, post-traumatic fatigue, and sensitivity to visual stimuli [21, 22]. This suggest that side-effects may be proportional to the proximity to the index traumatic or surgical. In our study, patients were included only after they had been stabilised and stepped down from intensive care and so the majority of patients received their first VR session on the second day after the event or later. While it is understood that the early initiation of intense and frequent neurorehabilitation is essential to maximise functional recovery, it may be that the balance between side effects of VR only becomes favourable after the first 48 h. Sub-group analyses between side effect rates in trauma and oncology patients were not performed as the study was underpowered. Neurotrauma and neurooncology patients may have differing side-effect profiles owing to the differing pathological processes. The location of tumour, surgery, and injury may also play a role and lesions in the visual or vomiting centres may have disproportional effects compared to lesions elsewhere. The low side-effect profile contributes to the feasibility and safety metrics defined in this study. Additional important observations are the zero intervention related infection and seizure rates. Cranial wounds are in close proximity to the head mounted display but with appropriate measures such as cleaning before and after each use and utilising a scub cap, patient comfort and hygine can be maintained. Seizures are relatively common in neurooncology, neurotrauma and post-neurosurgical population. Our findings may provide reassurance that the visual stimuli from the VR intervention does not increase seizure rates, as seen in this study. These findings are aligned with literature on adverse event rates in other studies, however because the majority of literature is from stroke or non-neurosurgical populations, our findings are an additional contribution to the evidence base [23].

Digital technologies that can accelerate recovery after surgery have been used in both pre- and post-operative settings [24, 25]. Awareness of Enhanced Recovery After Surgery (ERAS) protocols in neurosurgery is stronger in spinal than cranial neurosurgery, but neurosurgeons recognise that these protocols can reduce costs and lower intensive care admissions^26,27^. Combining ERAS protocols with relatively inexpensive digital technologies that augment recovery processes may scale benefits faster to more patients. The optimal integration of human-directed therapy and computer-directed therapy remains undefined, both in terms of therapy quantity and mode of delivery. VR therapy has demonstrated cost-effectiveness in cognitive therapy settings^28^. A study exploring VR in recovery after stroke proposes a randomised design where patients enter one of two groups, a control group receiving 100% (60 min) human therapy or an intervention group receiving 50% (30 min) VR and 50% (30 min) human therapy^29^. In this case, the quantity of therapy time is the same for both groups, yet the composition of therapy is different in comparing standard therapy with hybrid delivery of VR therapy. If the intervention group demonstrates non-inferiority, then it would be reasonable to expect the intervention to be cost-effective. Another approach could be to deliver additional therapy via VR alongside the standard amount of human-delivered therapy. This would increase the total ‘dose’ of therapy and may accelerate rehabilitation further without increasing human resource cost which can be greater than digital technology cost. Even with the current state-of-the-art VR technology, it seems unlikely that 100% of therapy could be delivered by computers alone safely and effectively. There are limits to what is technically possible and to what would be acceptable to patients. Importantly, there is also a need to consider the additional benefits of patient-clinician interactions that technology currently cannot fulfil. Further research should investigate the optimal combination of human resources and the most appropriate technologies in neurorehabilitation.

Strengths of this study include the use of this technology in a novel setting that has significant unmet need. Although neurosurgery is a comparatively small specialty, the disease burden from neurotrauma and neuro-oncology is significant globally - from both a financial and patient perspective. Other strengths include the use of validated data collection instruments and adherence to IDEAL feasibility methodological research frameworks. A further strength is the multidisciplinary working between neurosurgeons and neurorehabilitation therapists in the design and delivery of the study. Limitations include the lack of ability to infer any clinical benefit or sample sizes for future studies. One study in upper extremity stroke estimated a sample size of 96 in each arm to detect a clinically significant difference^30^. An important consideration for a future study would be choosing a patient-focused primary outcome, such as time to independence, time to discharge or time to return to work or a composite physical and cognitive functional metric. A further limitation is the heterogeneity in the study population. While the heterogeneity of the population allows investigation of VR feasibility across a wide proportion of disease areas that require neurorehabilitation, the small sample size limits both internal consistency and external validity. This means that the findings may not be fully generalisable, highlighting further the need for larger, more stratified studies. Another limitation is that this study only evaluated one VR hardware and software combination. Other technologies and applications may offer additional and different benefits that were not explored in this study. A final consideration is that all participants in this study had all bone flaps re-inserted and in place, and using the headset if no bone flap present may not be safe or feasible.

This study has implications for methodologists designing future research involving VR and similar technologies in acute neurosurgery patients. Neurosurgeons and neurorehabilitation therapists should explore further use cases for this technology and design powered, randomised clinical effectiveness studies with a focus on outcomes relevant to patients, including cost-effectiveness measures. Evidencing healthcare costs in this way would help provide support for investment and adoption strategies to improve rehabilitation services.

## Data Availability

The data that support the findings of this study are available from the corresponding author, WSB, upon reasonable request.
